# Salinity Is a Key Determinant for Soil Microbial Communities in a Desert Ecosystem

**DOI:** 10.1128/mSystems.00225-18

**Published:** 2019-02-12

**Authors:** Kaoping Zhang, Yu Shi, Xiaoqing Cui, Ping Yue, Kaihui Li, Xuejun Liu, Binu M. Tripathi, Haiyan Chu

**Affiliations:** a State Key Laboratory of Soil and Sustainable Agriculture, Institute of Soil Science, Chinese Academy of Sciences, Nanjing, China; b College of Resources and Environmental Sciences, Key Laboratory of Plant-Soil Interactions of the Ministry of Education, China Agricultural University, Beijing, China; c Key Laboratory of Biogeography and Bioresource in Arid Land, Xinjiang Institute of Ecology and Geography, Chinese Academy of Sciences, Urumqi, China; d Korea Polar Research Institute, Incheon, Republic of Korea; e University of Chinese Academy of Sciences, Beijing, China; University of Colorado Denver

**Keywords:** community assembly processes, community diversity, desert ecosystem, microbial phenotypes, salinity

## Abstract

Soil salinization is a growing environmental problem caused by both natural and human activities. Excessive salinity in soil suppresses growth, decreases species diversity, and alters the community composition of plants; however, the effect of salinity on soil microbial communities is poorly understood. Here, we characterize the soil microbial community along a natural salinity gradient in Gurbantunggut Desert, Northwestern China. Microbial diversity linearly decreased with increases in salinity, and community dissimilarity significantly increased with salinity differences. Soil salinity showed a strong effect on microbial community dissimilarity, even after controlling for the effects of spatial distance and other environmental variables. Microbial phylotypes (*n* = 270) belonging to *Halobacteria*, *Nitriliruptoria*, [*Rhodothermi*], *Gammaproteobacteria*, and *Alphaproteobacteria* showed a high-salinity niche preference. Out of nine potential phenotypes predicted by BugBase, oxygen-related phenotypes showed a significant relationship with salinity content. To explore the community assembly processes, we used null models of within-community (nearest-taxon index [NTI]) and between-community (βNTI) phylogenetic composition. NTI showed a significantly negative relationship with salinity, suggesting that the microbial community was less phylogenetically clustered in more-saline soils. βNTI, the between-community analogue of NTI, showed that deterministic processes have overtaken stochastic processes across all sites, suggesting the importance of environmental filtering in microbial community assembly. Taken together, these results suggest the importance of salinity in soil microbial community composition and assembly processes in a desert ecosystem.

**IMPORTANCE** Belowground microorganisms are indispensable components for nutrient cycling in desert ecosystems, and understanding how they respond to increased salinity is essential for managing and ameliorating salinization. Our sequence-based data revealed that microbial diversity decreased with increasing salinity, and certain salt-tolerant phylotypes and phenotypes showed a positive relationship with salinity. Using a null modeling approach to estimate microbial community assembly processes along a salinity gradient, we found that salinity imposed a strong selection pressure on the microbial community, which resulted in a dominance of deterministic processes. Studying microbial diversity and community assembly processes along salinity gradients is essential in understanding the fundamental ecological processes in desert ecosystems affected by salinization.

## Abstract (precis)

Belowground microorganisms are indispensable components for nutrient cycling in desert ecosystems, and understanding how they respond to increased salinity is essential for managing and ameliorating salinization. Our sequence-based data revealed that microbial diversity decreased with increasing salinity, and certain salt-tolerant phylotypes and phenotypes showed a positive relationship with salinity. Using a null modeling approach to estimate microbial community assembly processes along a salinity gradient, we found that salinity imposed a strong selection pressure on the microbial community, which resulted in a dominance of deterministic processes. Studying microbial diversity and community assembly processes along salinity gradients is essential in understanding the fundamental ecological processes in desert ecosystems affected by salinization.

## INTRODUCTION

About 1/10 of the total dry land surface on the earth suffers from salinization (1), and salinized areas are increasing due to low precipitation, high surface irrigation, and poor agricultural management. Soil salinity acts as an influential environmental stressor coupled with limitation of water availability and high intracellular concentrations of ions that are toxic to metabolic activities (2). High salinity in soil suppresses plant growth, decreases plant photosynthetic capacities (3), and poses a strong influence on the composition, distribution, and diversity of plant communities (4). Belowground microorganisms are crucial for carbon decomposition and nutrient cycling, but the potential effect of soil salinity on belowground microbial communities is poorly understood.

Initially, the effect of salinity on soil microorganisms was studied using traditional approaches, such as soil respiration, microbial biomass, and microbial enzymatic activities (5). Most field and laboratory experiments showed an adverse effect of salinity on soil microbial biomass, respiration (6–9), and enzymatic activities (1). However, a microcosm experiment demonstrated that total microbial biomass and bacterial biomass evaluated by phospholipid fatty acid (PLFA) were not affected by soil salinity (10), and a field study with a relatively modest salinity range in tidal wetlands showed that the activities of carbon-degrading extracellular enzymes and alkaline phosphatase activities were stimulated by salinity (11). The inconsistent responses probably came from the pools of microbial phylotypes present in different experiment sites and their differences in salt tolerance. With the development of next-generation sequencing technologies, several studies have recently investigated the shifts in community structures of microbial phylotypes associated with salinity in saline sediments and soils (2, 12–14). However, the extent to which the changes in salinity levels are the main driver for microbial community divergence is still debatable. For example, a study explored both soil and sediment samples collected along a 140-m transect from the hypersaline lake La Sal del Rey, and the variance of the microbial community was shaped by oxygen, carbon substrates, and pH rather than salinity (12), while sediment samples collected in Qinghai-Tibetan lakes showed that salinity was a key factor in shaping microbial diversity and community structure (13). A better understanding of how microorganisms respond to a natural salinity gradient is important in predicting the vulnerability of desert ecosystems to environmental change.

Although salinity had been demonstrated to be the most important factor to affect microbial distribution at a global scale (15, 16), no previous study has focused explicitly on microbial community assembly processes along natural salinity gradients. The importance of understanding community assembly processes is broadly recognized in microbial ecology (17–19), and the assembly of microbial communities is known to be influenced by both deterministic and stochastic processes (20, 21). Deterministic processes refer to habitat filtering or biotic interactions such as mutualism, commensalism, and parasitism, while stochastic processes refer to random demographic changes in mortality and passive dispersal (18, 22). By examining deviations from null model expectations, changes in the relative importance of deterministic and stochastic processes for microbial communities can be investigated (23). Recent studies investigated community assembly processes along aridity (21) and pH (24, 25) gradients, but little is known about microbial community assembly processes along a salinity gradient.

The Gurbantunggut Desert, part of the Dzungarian Basin in northern Xinjiang, is the second largest desert in China. Because unfavorable environment conditions in deserts limit plant growth (26, 27), soil microbial communities in this ecosystem are less affected by plants. In addition, this region includes natural salinity gradients, which could provide an ideal simplified environment to study the effects of salinity on soil microbial communities. The goals of this study were to (i) determine how the diversity and composition of the microbial community vary along natural salinity gradients in desert ecosystems, (ii) investigate how salinity affects soil microbial phylotypes and phenotypes, and (iii) explore how salinity affects soil microbial community assembly processes.

## RESULTS

A total of 4,244,827 16S rRNA V4 region gene sequences were obtained across 120 soil samples. From the sequencing data, 15,147 operational taxonomic units (OTUs) were annotated at 97% identity. The dominant microbial phyla included *Actinobacteria* (∼41.32%), *Proteobacteria* (∼24.21%), *Bacteroidetes* (∼5.08%), *Chloroflexi* (∼5.38%), and *Firmicutes* (∼6.28%), accounting for more than 80% of the total sequences (see Fig. S2 in the supplemental material).

**FIG 1:**
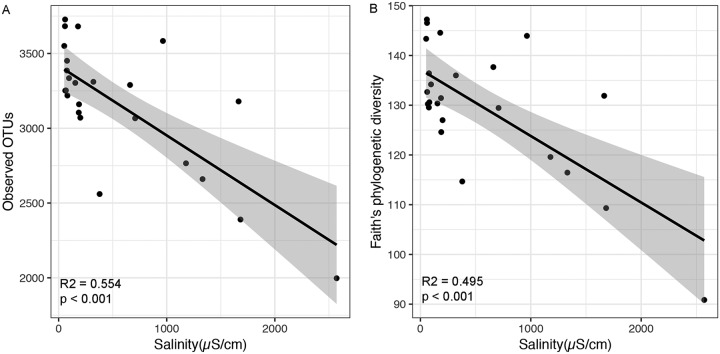
Relationship between soil salinity and observed OTUs (A) and Faith’s phylogenetic diversity (B).

We first explored the relationship between microbial alpha diversity (observed OTUs and Faith’s phylogenetic diversity) and 15 environmental variables (Table S1). Using stepwise multiple-regression model analysis, we found that salinity was consistently the best predictor for both observed OTUs and phylogenetic diversity, explaining 22.5% and 18.2% of the variation in the number of observed OTUs and Faith’s phylogenetic diversity, respectively (Table 1). Furthermore, salinity had a strong negative linear relationship with observed OTUs and Faith’s phylogenetic diversity (Fig. 1).

**TABLE 1:** Results of stepwise multiple-regression models using observed OTUs and phylogenetic diversity as response variables^*a*^

Response variable	*R*^2^ (%)	Predictor variable	*F*	*P*
Observed OTUs	46.17	Salinity	53.14	<0.001
		WC	26.36	<0.001
		pH	18.59	<0.001
		SOC	8.23	0.004
		P	2.04	0.156
		NO_3_^−^	0.37	0.542
		DTN	0.35	0.556

PD	43.71	Salinity	40.50	<0.001
		WC	30.76	<0.001
		pH	12.29	<0.001
		P	7.01	0.009
		SOC	6.87	0.01

a PD, phylogenetic diversity; WC, soil water content; SOC, soil organic carbon; DTN, dissolved total nitrogen; P, available phosphorus.

10.1128/mSystems.00225-18.4 TABLE S1 Summary statistics of environmental variables. CV, coefficient of variation; range, minimum to maximum; WC, soil water content; SOC, soil organic carbon; DOC, dissolved organic carbon; DTN, dissolved total nitrogen; TN, total nitrogen; P, available phosphorus; K, available potassium; TEM, average air temperature in May 2016; EVI, enhanced vegetation index. Download Table S1, XLSX file, 0.03 MB. Copyright © 2019 Zhang et al. 2019 Zhang et al. This content is distributed under the terms of the Creative Commons Attribution 4.0 International license.

**FIG 2:**
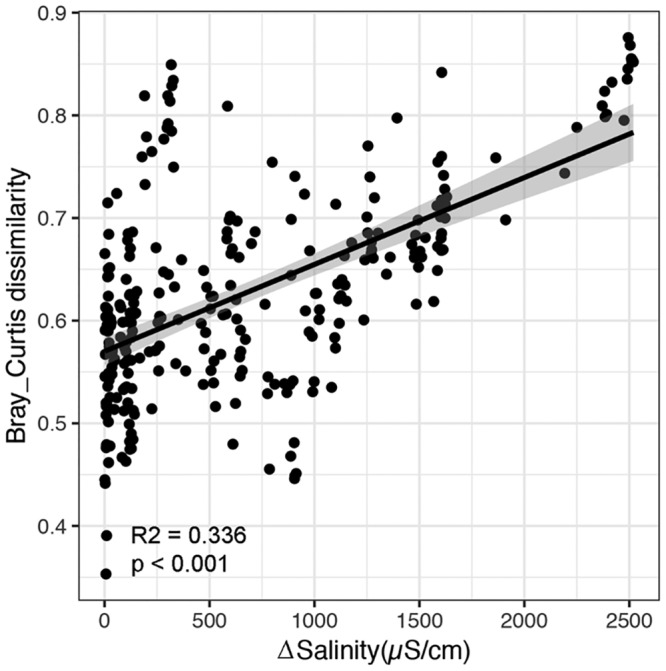
Relationship between Bray-Curtis dissimilarity and differences in soil salinity.

Distance-based multivariate linear model (DistLM) analysis showed that salinity was the most important factor that determined microbial community structure and explained 9.35% of the total variations in microbial community structure (Table S2). Furthermore, multivariate regression tree (MRT) analysis was used to probe the effects of environmental variables on microbial community structure. Even though all 15 measured environmental variables were included in the analysis, only salinity content split the tree and divided samples into three salinity gradients (Fig. S3). Microbial Bray-Curtis dissimilarity also showed a significant negative relationship with differences in soil salinity (*R*^2^ = 0.336; *P* < 0.001) (Fig. 2), which indicates that the larger the salinity difference between two sites, the more dissimilarity between the microbial community structure in those two sites. As geographic distance is also an important factor to elicit variation in microbial community structure, a partial Mantel test was used to estimate the effect of salinity distance on microbial community structure after controlling for spatial distance and other environmental distances, excluding salinity. Even though both salinity and geographic distance had significant effects on microbial community structure, the effect of salinity was stronger than that of geographic distance (Table 2). Together, these observations strongly suggested that salinity was a key factor in shaping the structure and diversity of a desert soil microbial community.

**TABLE 2:** Partial Mantel test results showing comparisons between microbial community dissimilarity, βNTI, and a one-distance matrix while controlling for the other two distance matrices

Test	Parameter	Effect of^*a*^:		
		Salinity.dist controlling for Env.dist (excluding salinity) + Geo.dist	Env.dist (excluding salinity) controlling for Geo.dist + salinity.dist	Geo.dist controlling for Env.dist (excluding salinity) + salinity.dist
Bray-Curtis dissimilarity	*r*	0.465	0.021	0.1524
	*P*	0.001	0.332	0.001

βNTI	*r*	0.072	−0.092	−0.084
	*P*	0.057	0.967	0.999

a Salinity.dist, salinity dissimilarity based on Euclidean distance; Env.dist (excluding salinity), all the measured variables except salinity distance based on Euclidean distance; Geo.dist, geographic distance.

10.1128/mSystems.00225-18.5 TABLE S2 Results of distance-based multivariate linear models (DistLM) for soil microbial community composition showing the percentage of variance explained by environmental variables. Values in boldface type indicate a significant correlation (*P* < 0.05). Ex. Var. (%), percentage of variance explained by environmental variables; Cum. (%), cumulative percentage of variance explained; DOC, dissolved organic carbon; TN, total nitrogen; K, available potassium; TEM, air temperature; EVI, enhanced vegetation index. Download Table S2, XLSX file, 0.03 MB. Copyright © 2019 Zhang et al. 2019 Zhang et al. This content is distributed under the terms of the Creative Commons Attribution 4.0 International license.

**FIG 3:**
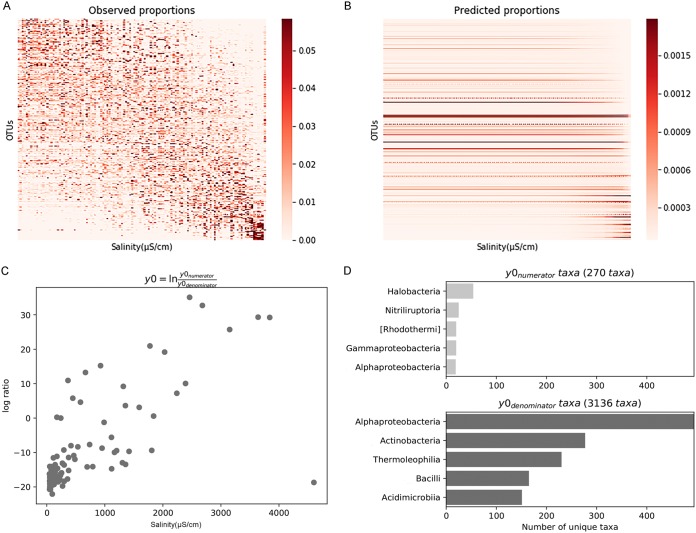
Balance tree estimated by genies analysis showing niche differentiation of soil microbial OTUs. (A) Heat map showing observed OTU proportions sorted by salinity from 46.2 μS/cm to 4,601 μS/cm. (B) Heat map showing predicted OTU proportions from ordinary least-squares linear regression on balances sorted by salinity. (C) Log ratio of proportions of OTUs with a low-salinity niche preference to proportions of OTUs with a high-salinity niche preference along a salinity gradient. *y*0_denominator_ represents low-salinity OTUs with salinity ranges from 46.2 μS/cm to 535 μS/cm, and *y*0_numerator_ represents high-salinity OTUs with salinity ranges from 535 μS/cm to 4,601 μS/cm. (D) Number of OTUs belonging to *y*0_denominator_ and *y*0_numerator_ sorted to the class level.

**FIG 4:**
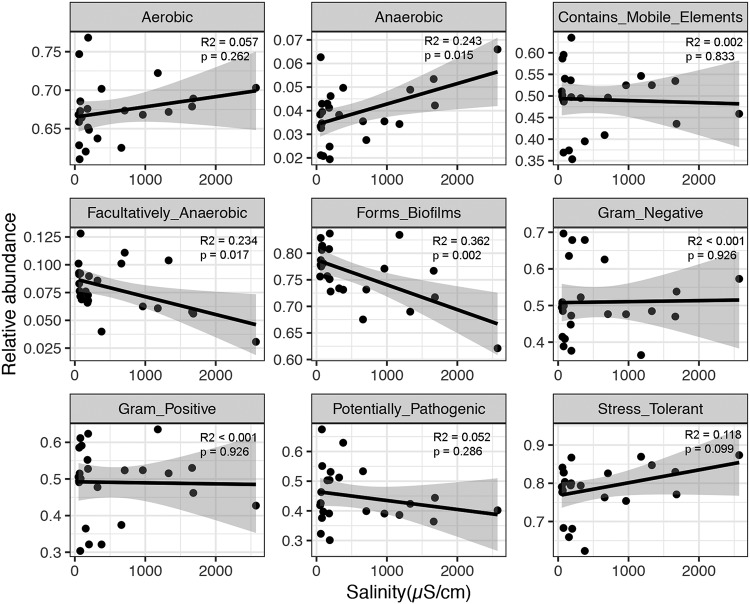
Relationship between soil salinity and relative abundances of nine potential phenotypes predicted by BugBase.

Salinity has been found to be a major driver of microbial diversity and composition at the community level, which inspired us to investigate the effect of salinity on soil microorganisms at finer taxonomic levels such as phylotypes. The balance tree approach showed well-defined niche differentiation of microbial OTUs along a salinity gradient (Fig. 3A and B). The high-salinity OTUs (535 to 4,601 μS/cm) were gradually overtaken by low-salinity OTUs (46.2 to 535 μS/cm) as the salinity increased, forming a linear trend by the top balance of the tree (Fig. 3C). To extract taxon information from the top balance of the tree, 270 taxa belonging to *Halobacteria*, *Nitriliruptoria*, [*Rhodothermi*], *Gammaproteobacteria*, and *Alphaproteobacteria* were found to be more abundant in high-salinity sites, while 3,136 taxa belonging to *Alphaproteobacteria*, *Actinobacteria*, *Thermoleophilia*, *Bacilli*, and *Acidimicrobiia* were more abundant in low-salinity sites (Fig. 3D). Using BugBase, we predicted nine potential phenotypes, including aerobic, anaerobic, containing mobile elements, facultatively anaerobic, biofilm forming, Gram negative, Gram positive, potentially pathogenic, and stress tolerant. Among all the phenotypes, the relative abundance of the anaerobic phenotype showed a significant positive relationship with salinity, and the relative abundance of the stress-tolerant phenotype showed a marginally significant (*P* < 0.1) positive relationship with salinity, while the relative abundance of facultative anaerobic and biofilm-forming phenotypes displayed a significantly negative relationship with salinity (Fig. 4).

**FIG 5:**
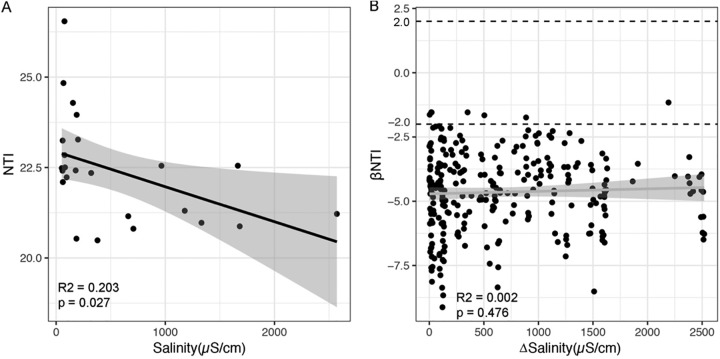
Relationship between soil salinity and within-community NTI (A) and between-community βNTI (B) of a microbial community.

Furthermore, to figure out how salinity influenced microbial community assembly processes, we used within-community (nearest-taxon index [NTI]) and between-community (βNTI) null models. We found a significant negative relationship between NTI and salinity (Fig. 5A), indicating that the increase in salinity decreased the extent of phylogenetic clustering in the microbial community. Even though there was no significant relationship between βNTI and the difference in salinity, almost all the βNTI values were below −2, which implied a dominant role of environment factors in the microbial community.

## DISCUSSION

The first objective of this study was to explore the effect of salinity on microbial community diversity and community structure in a desert ecosystem. In this study, we used amplicon sequencing to investigate the salinity effect on a microbial community and found that observed OTUs and Faith’s phylogenetic diversity had significant negative relationships with salinity (Fig. 1). The possible explanation for this negative effect could be attributed to the fact that the accumulation of salt in soils elevates the extracellular osmolarity (5, 28), and many microorganisms that fail to adapt to osmotic stress may die or become inactive, thus reducing microbial alpha diversity. Variation in soil microbial community structure was also mainly explained by salinity in this study, which is consistent with the results found in estuarine and marine environments (29–31). However, a study investigating soil and sediment microbial communities near a hypersaline lake with salinity ranges from 34.2 mS/cm to 123 mS/cm found that shifts in microbial community were highly related to the site water content, nutrient concentrations, and pH rather than salinity (12). The contrasting results reported in the previous study might be due to local-scale sampling in an already salt-rich environment.

The variation in microbial community composition along the salinity gradient reported in this study (Fig. 2) had implications for species sorting, with more-salt-tolerant species replacing less-salt-tolerant ones. Those microorganisms that thrive in high-salinity environments often apply two strategies to balance the osmotic potential of the cytoplasm (28). One is the “salt-in” strategy, which involves taking up ions, such as predominantly potassium ions. This strategy is often used by some halophiles, such as *Halobacteriaceae*, *Salinibacter*, and fermentative *Halanaerobiales*. Hence, it was reasonable to find more *Halobacteria* in soils with high salinity (Fig. 3D). Consistently, *Halobacteriaceae* have been shown to be prevalent in saline soils (32), lake sediments (33), and marine environments (34). The other strategy is the “low-salt-in” strategy, which involves accumulating low-molecular-weight organic compounds (e.g., amino acids and carbohydrates) within the cell to exclude salt from the cell (35). Previous culture-independent studies detected dominant halophilic and halotolerant taxa affiliated with the bacterial phyla *Proteobacteria*, *Actinobacteria*, *Bacteroidetes*, and *Gemmatimonadetes* (36, 37). The microbial taxa belonging to *Proteobacteria*, *Bacteroidetes*, *Actinobacteria*, and *Halobacteria* detected in this study have a high-salinity niche preference (Fig. 3), and these taxa may act as potential biomarkers for a high-salinity-tolerant community. Furthermore, to reveal the responses of functional traits to increased salinity, BugBase was used to predict potential phenotypes. The oxygen-related phenotypes showed a significant relationship with salinity (Fig. 4). High salinity has been demonstrated to elicit dispersion of soil particles (38); thus, it was reasonable that these oxygen-related phenotypes changed, as oxygen availability could be affected by the dispersion of soil particles (39).

Salinity is not the only pressure for microorganisms in a desert, which is often combined with low water availability and high pH (5). Soil moisture was found to be the second important factor affecting both community diversity and structure in this study (Table 1; see also Table S2 in the supplemental material). The microbial cell contains nearly 70% water, and soil water content is an important factor that determines a microbial community, as it exerts strong control over gaseous and liquid diffusion of microbial resources within soil (40). A short-term microcosm experiment that combined salinity and drying-rewetting processes together found that inhibition of bacterial growth and respiration by reduced moisture was exacerbated by the accumulation of salinity content (9), indicating a more-severe effect on the microbial community than only high salinity.

The dramatically low microbial diversity in high-salinity sites in the present study indicates habitat filtering, which was supported by all positive NTI values (Fig. 5A). The positive NTI values suggest that the communities were more phylogenetically clustered than expected by chance (41), reflecting an environmental selection pressure on the microbial community to form a nonrandom community pool (42). Even though salinity had a strong selective pressure on the microbial community, it should be noted that NTI decreased with increasing salinity, which means that microbial community assembly was less phylogenetically clustered in more-saline soils. Knowing that salinity is a major determinant of the microbial community and that only taxa well adapted to high salt concentrations are able to prevail in high-salt environments (28), the strength of phylogenetic clustering is expected to increase when the environment is suitable for only a subset of microorganisms (43). In a previous study, it was found that the extent of soil bacterial phylogenetic clustering was greater in more-acidic and more-alkaline soils (25). The contrasting results obtained in this study could be explained in two ways. First, although microorganisms live in high-salt environments, this does not necessarily mean that only closely related taxa coexist in such peculiar environments. For example, a previous review exploring the diversity of *Archaea* in hypersaline systems found that this high-salinity habitat harbors a phylogenetically diverse group of *Archaea* possessing different metabolic pathways (44). Second, the limited resource availability in high-salinity sites because of sparse plant growth (5) could result in the competitive exclusion of some closely related taxa, which acts as a signal for overdispersed phylogeny (42). In this study, the strong effect of salinity on microbial community structure and assembly could be explained by the higher relative importance of deterministic processes, and the dominant role of deterministic processes was tested by βNTI (Fig. 5B).

In conclusion, we characterized a soil microbial community by sequencing the 16S rRNA genes in a desert ecosystem along a natural soil salinity gradient. Our results provide strong evidence for a salinity effect on microbial community composition and assembly, which will shed light on how desert ecosystems may respond to ongoing salinization. To move forward our understanding of the dynamics of ecosystems under these severe salinization conditions, future effort should be made to build extensive data sets that can be used to explore the general rules of how microbes respond to increasing salinity.

## MATERIALS AND METHODS

### Soil sample collection.

The sampling sites were along an east-to-west transect in the Gurbantunggut Desert, Xinjiang, Northwestern China, at 44.21°N to 45.51°N and 83.16° to 91.77°E (see Fig. S1 in the supplemental material). This region has a temperate continental arid climate, with a mean annual temperature range from 6.4°C to 7.7°C and mean annual rainfall from 102 to 167.4 mm during 2011 to 2013 (45). Twenty-four sampling sites were selected along the 682.8-km transect in 5 to 13 May 2016. At each sampling site, five 1-m by 1-m quadrats were selected as replicates within a 500-m by 500-m quadrat, and the five plots were about 300 m apart from each other within the 500-m by 500-m quadrat. To reduce the effects of soil heterogeneity, we also collected five soil samples within the 1-m by 1-m quadrats and composited them to make one soil sample per quadrat (Fig. S1). A total of 120 topsoil (0- to 15-cm) samples were collected by drill, and all samples were stored on ice in the field and immediately transported to the laboratory. After sieving through a 2-mm mesh, each soil sample was divided into two parts, with half stored at 4°C for soil biogeochemical property analyses and half stored at −20°C for DNA extraction. In addition, for each sampling site, we collected data on average air temperature (TEM), rainfall, and the enhanced vegetation index (EVI) on May 2016 from the meteorological data platform of China (http://data.cma.cn/site/index.html).

10.1128/mSystems.00225-18.1 FIG S1 Locations of sampling sites in Gurbantunggut Desert, quadrat sets in each sampling site, and one of the sampling quadrats. Download FIG S1, PDF file, 0.4 MB. Copyright © 2019 Zhang et al. 2019 Zhang et al. This content is distributed under the terms of the Creative Commons Attribution 4.0 International license.

10.1128/mSystems.00225-18.2 FIG S2 Pie showing soil-dominant prokaryotic phyla (relative abundance of >1%) across all the sampling sites. Download FIG S2, PDF file, 0.1 MB. Copyright © 2019 Zhang et al. 2019 Zhang et al. This content is distributed under the terms of the Creative Commons Attribution 4.0 International license.

10.1128/mSystems.00225-18.3 FIG S3 Multivariate regression tree analyses of microbial community structure based on Bray-Curtis distance with environmental variables. The tree was done using the “lse” method with 999 cross-validations. Download FIG S3, PDF file, 0.06 MB. Copyright © 2019 Zhang et al. 2019 Zhang et al. This content is distributed under the terms of the Creative Commons Attribution 4.0 International license.

### Analysis of soil biogeochemical properties.

Soil pH was measured by using an E20-FiveEasy pH meter (Mettler Toledo, Germany), and soil electrical conductivity, the indicator of soil soluble salt, was determined by using an electric conductometer. Both soil measurements were made using a soil-water suspension (5:1 mixture of deionized water-fresh soil) after shaking for 30 min. Soil moisture was determined gravimetrically at ∼105°C for 6 h. Dissolved total nitrogen (DTN), nitrate (NO_3_^−^-N), and ammonium (NH_4_^+^-N) were extracted by adding 5 g fresh soil to 50 ml of a 2 M KCl solution, and dissolved organic carbon (DOC) was extracted with 50 ml of deionized water. After shaking for 1 h and standing for 1 h, the supernatant was filtered through glass fiber filters (Fisher G4, 1.2-μm pore space). The concentrations of NO_3_^−^-N, NH_4_^+^-N, and DTN were determined using a continuous-flow analytical system (San^++^ system; Skalar, Holland). DOC was determined by using a carbon nitrogen analyzer (Multi N/C 3000; Analytik Jena, Germany). Available phosphorus (P) was extracted with a 0.5 M NaHCO_3_ solution and measured by the Mo-Sb colorimetric method. Available potassium (K) was extracted with 1 M ammonium acetate (NH_4_OAc) and measured by the flame spectrophotometry method. Soil organic matter (SOM) was measured by the K_2_Cr_2_O_7_-H_2_SO_4_ oxidation method, and total nitrogen (TN) was measured by the Kjeldahl method.

### Soil DNA extraction and 16S rRNA sequencing.

For each sample, DNA was extracted from 0.5 g fresh soil using a Fast DNA spin kit for soil (MP Biomedicals, Santa Ana, CA) according to the manufacturer’s instructions. DNA was then quantified using a Nanodrop 1000 spectrophotometer (Thermo Scientific, Wilmington, DE) and stored at −20°C before sequencing. Primers 515F (GTGCCAGCMGCCGCGG) and 806R (GGACTACHVGGGTWTCTAGGTWTCTAAT) were used to amplify the V4 hypervariable region of the 16S rRNA gene in both bacteria and archaea (46). PCR was carried out in a 30-µl reaction mixture volume with 15 µl Phusion high-fidelity PCR master mix (New England Biolabs), 0.2 µl forward and reverse primers, and ∼10 ng template DNA. Thermal cycling was carried out at 98°C for 1 min, followed by 30 cycles at 98°C for 10 s, 50°C for 30 s, and 72°C for 30 s. High-throughput sequencing was performed on an Illumina HiSeq platform (Illumina, Inc., USA), and 250-bp paired-end reads were generated.

### Data analysis.

The barcoded forward and reverse reads of 16S rRNA genes were merged by using FLASH (47). Paired-end reads were assigned to each sample based on unique barcodes and analyzed in QIIME1.9.0 using default settings (48). Sequences were clustered into operational taxonomic units (OTUs) by UCLUST with a 97% similarity threshold using QIIME’s pick_open_reference_otus.py script and the Greengenes database (13-8 release) as a reference (49). Low-abundance OTUs were eliminated from the OTU table when the number of counts across all samples was <10. QIIME’s core_diversity_analyses.py script was used to compute alpha and beta diversity values, and all samples were rarefied to 27,000 sequences per sample.

Stepwise multiple-regression analysis was conducted to identify the main predictors of microbial diversity (observed OTUs and phylogenetic diversity) among the measured environmental variables. The distance-based multivariate linear model (DistLM) was also used to test the correlation between environmental variables and microbial community structure based on Bray-Curtis distance. The contribution of each environmental variable was assessed using DISTLM_forward3 (50). Multivariate regression tree (MRT) analysis was also performed to detect relationships between microbial community structure and all measured environmental variables (51). A total of 1,000 cross-validations using the “lse” method were used to decrease the complexity of the tree to identify the main predictors of microbial community structure. MRT analysis was conducted using the mvpart package in R. Niche differentiation of microbial phylotypes was detected by a more precise method, gneiss in QIIME2 (52). The method uses the concept of a balance tree to infer changes of microbial subcommunities to evaluate niche differentiation rather than changes in individual species based on proportion. Gradient clustering was applied to group microbes into their preferred habitat, ilr-transform was used to compute the isometric log ratios between groups, and ordinary least-squares analysis was used to calculate the balances of microbial community profiles. OTUs with fewer than 120 reads were filtered in the gneiss analysis to avoid clustering errors. BugBase, an organism-level prediction algorithm, can be used to predict biologically interpretable phenotypic traits, such as Gram status, oxygen requirements, and biofilm formation (53). A Web application version of BugBase (http://bugbase.cs.umn.edu) was used to obtain phenotypic information based on 16S rRNA gene sequences in this study.

The nearest-taxon index (NTI) were used to evaluate the phylogenetic community assembly on a within-community scale, and high or positive values represent clustering of taxa across the overall phylogeny, while low or negative values indicate overdispersion of taxa across the phylogeny (41). The value of NTI is equivalent to −1 times the standardized effect size of MNTD (mean nearest-taxon distance), and the standardized effect size of MNTD was calculated by comparing observed phylogenetic relatedness to the expected pattern under the “taxa.lables” null model with 999 randomizations in the “picante” R package. The abundance-weighted βMNTD was calculated to infer community phylogenetic turnover between communities using Phylocom software (54). Next, a between-community null modeling approach was applied to infer community assembly processes by calculating the β-nearest-taxon index (βNTI). βNTI represents the deviation between the observed βMNTD and the expected βMNTD. As the expected βMNTD represents the dominance of stochastic processes, the value of βNTI can be used to infer the dominance of stochastic and deterministic processes. βNTI pairwise comparisons falling within the null distributions (−2 < βNTI < 2) indicate a dominance of stochastic processes, whereas proportions of pairwise comparisons for which the βNTI is more than 2 or less than −2 indicate a dominance of deterministic processes (55). A partial Mantel test with Pearson correlation was used to estimate the relationship between βNTI, microbial community dissimilarity (Bray-Curtis distance), and a one-explanation matrix (such as soil salinity distance based on Euclidean distance, spatial distance, or environmental distance, excepting salinity based on Euclidean distance) after controlling for the other two matrices.

### Data availability.

All sequencing data associated with this study have been deposited at the NCBI Sequence Read Archive (SRA) under project accession number SRP112798.
