# Supplementary figures and images for: Salinity Is a Key Determinant for Soil Microbial Communities in a Desert Ecosystem
Source: mSystems. 2019 Feb 12;4(1):e00225-18. doi: 10.1128/mSystems.00225-18 (PMC6372838; doi:10.1128/mSystems.00225-18)

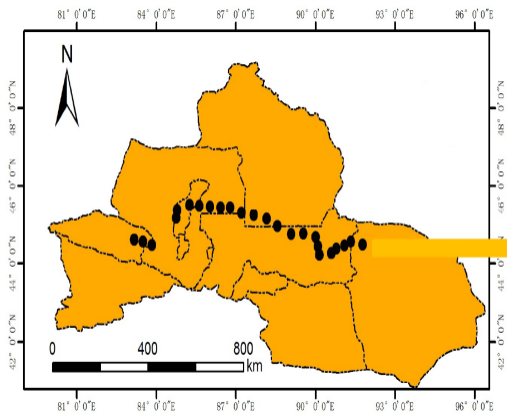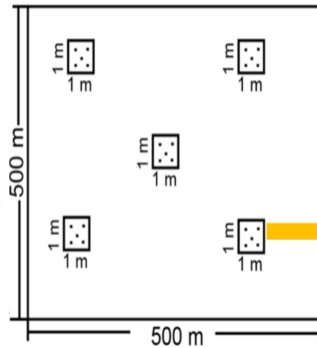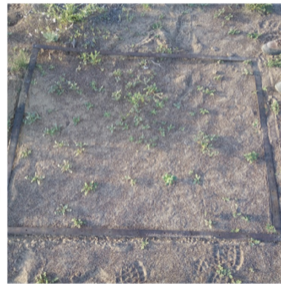

Supplement: FIG S1 [file mSystems.00225-18-sf001.pdf]

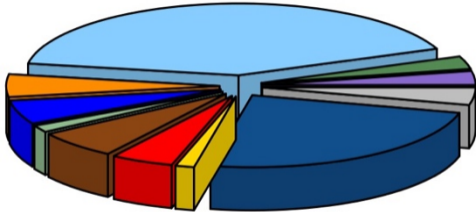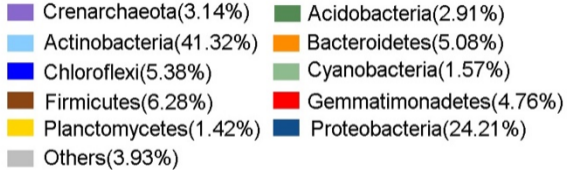

Supplement: FIG S2 [file mSystems.00225-18-sf002.pdf]
