# Supplementary material for: Salinity Is a Key Determinant for Soil Microbial Communities in a Desert Ecosystem
Source: mSystems. 2019 Feb 12;4(1):e00225-18. doi: 10.1128/mSystems.00225-18 (PMC6372838; doi:10.1128/mSystems.00225-18)
Supplement: FIG S3 [file mSystems.00225-18-sf003.pdf]

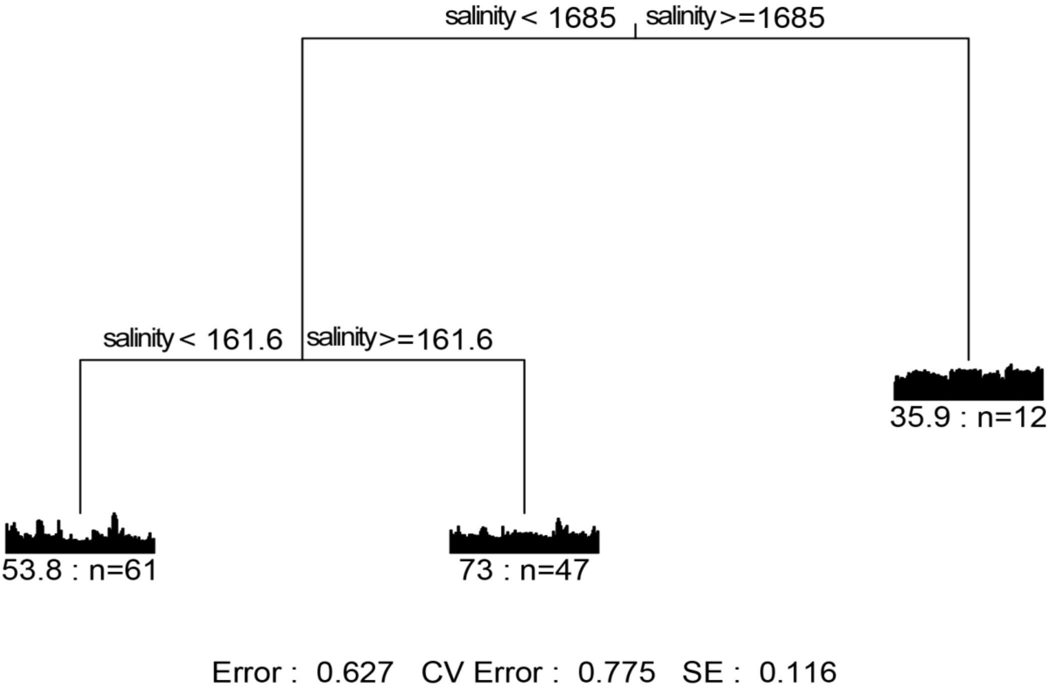
salinity< 1685
salinity>=1685
salinity< 161.6
salinity>=161.6
35.9 : n=12
53.8 : n=61
73 : n=47
Error : 0.627 CV Error : 0.775 SE : 0.116
